# From Stroke to Dementia: a Comprehensive Review Exposing Tight Interactions Between Stroke and Amyloid-β Formation

**DOI:** 10.1007/s12975-019-00755-2

**Published:** 2019-11-28

**Authors:** Romain Goulay, Luis Mena Romo, Elly M. Hol, Rick M. Dijkhuizen

**Affiliations:** 1 Biomedical MR Imaging and Spectroscopy Group, Center for Image Sciences, University Medical Center Utrecht, Utrecht University, Yalelaan 2, 3584 CM Utrecht, The Netherlands; 2 Department of Neurology, University Hospital Sant Juan Despi Moises Broggi, Barcelona, Spain; 3 Department of Translational Neuroscience, UMC Utrecht Brain Center, University Medical Center Utrecht, Utrecht University, Utrecht, The Netherlands; 4 Department of Neuroimmunology, Netherlands Institute for Neuroscience, an institute of the Royal Netherlands Academy of Arts and Sciences, Amsterdam, The Netherlands

**Keywords:** Stroke, Alzheimer’s disease, Dementia, Cerebral amyloid angiopathy, Beta-amyloid

## Abstract

Stroke and Alzheimer’s disease (AD) are cerebral pathologies with high socioeconomic impact that can occur together and mutually interact. Vascular factors predisposing to cerebrovascular disease have also been specifically associated with development of AD, and acute stroke is known to increase the risk to develop dementia.

Despite the apparent association, it remains unknown how acute cerebrovascular disease and development of AD are precisely linked and act on each other. It has been suggested that this interaction is strongly related to vascular deposition of amyloid-β (Aβ), i.e., cerebral amyloid angiopathy (CAA). Furthermore, the blood–brain barrier (BBB), perivascular space, and the glymphatic system, the latter proposedly responsible for the drainage of solutes from the brain parenchyma, may represent key pathophysiological pathways linking stroke, Aβ deposition, and dementia.

In this review, we propose a hypothetic connection between CAA, stroke, perivascular space integrity, and dementia. Based on relevant pre-clinical research and a few clinical case reports, we speculate that impaired perivascular space integrity, inflammation, hypoxia, and BBB breakdown after stroke can lead to accelerated deposition of Aβ within brain parenchyma and cerebral vessel walls or exacerbation of CAA. The deposition of Aβ in the parenchyma would then be the initiating event leading to synaptic dysfunction, inducing cognitive decline and dementia. Maintaining the clearance of Aβ after stroke could offer a new therapeutic approach to prevent post-stroke cognitive impairment and development into dementia.

## Introduction

Alzheimer’s disease (AD) and stroke are severe cerebral pathologies with a very high socioeconomic impact and burden for society. These pathologies can occur together, may even interact [1, 2], and both contribute to dementia. Vascular factors predisposing to stroke and cerebrovascular disease have been associated with dementia and more specifically with the enhancement of amyloid-β (Aβ) deposition [3]. One of the most common predisposing factors is cerebral amyloid angiopathy (CAA). CAA is a vascular disease involving Aβ depositions in the smooth muscle layer of vessel walls, associated with cognitive decline and both ischemic and hemorrhagic stroke [4, 5]. CAA appears to occur preferably in arteries within the brain, such as leptomeningeal and cortical arteries [6], but it may also affect cerebral capillaries [7]. CAA is increasingly diagnosed during life thanks to the development of advanced imaging technology enabling detection of cerebral micro-lesions (i.e., micro-infarcts or micro-bleeds) [8]. The prevalence of CAA in AD patients is about 78% [9, 10], and CAA might also be the critical factor linking stroke and dementia [11].

The recently introduced concept of the glymphatic system, characterized as a cerebral drainage system dependent on water channels located on astrocyte end-feet [12] and lymphatic vessels [13], may be closely involved in the pathophysiology of cerebrovascular and neurodegenerative diseases. For example, Aβ drainage via the cerebral perivascular space has been found to be impaired with aging leading to increase in Aβ deposits in an AD mouse model [14–16]. Furthermore, it has been reported that this perivascular pathway is impaired after both hemorrhagic and ischemic stroke, as observed in rodents and non-human primates [17, 18]. Abnormal perivascular space integrity, potentially correlated with malfunction of the glymphatic system and Aβ drainage, could therefore be considered as a possible mechanism explaining the link between CAA, stroke, and dementia [6, 16, 19].

Several earlier reviews have focused on the relationship between CAA and stroke or the relationship between CAA and dementia [4, 5, 20]. Different approaches exist to prevent or treat CAA (e.g., by inhibition of Aβ production, enhancement of Aβ clearance, or protection of vessels from the toxic effects of Aβ) [8, 21]. Even if the benefit of these strategies is uncertain in Alzheimer’s disease, these approaches could still be effective to prevent post-stroke dementia. However, one of the main difficulties in the design of clinical trials on CAA is the lack of consensus on the existence (and value) of biomarkers for the evaluation of treatment efficiency [22]. Assessment of the mechanisms connecting CAA to stroke and dementia could lead to original biomarkers for diagnostic and therapeutic evaluation in preclinical and clinical settings. In this review, we describe the different mechanisms that may underlie the interaction between stroke and dementia, with a specific focus on CAA and the glymphatic system, and we propose new possible clinical biomarkers and therapeutic strategies related to this relationship.

## Production, Physiological Roles, and Pathophysiological Consequences of Aβ

Amyloid precursor protein (APP) is a transmembrane protein that is mostly produced in the central nervous system by neurons [23] and synthesized by non-neuronal cells, such as smooth muscle cells in artery walls [24, 25], astrocytes, and oligodendrocytes. The amyloidogenic pathway of APP processing involves several successive cleavages. First, β-secretase (β-APP cleaving enzyme (BACE)) processes β-site APP into endosomes [26] to generate the soluble APP-β peptide, which is released into the extracellular space [27]. Following BACE-1 cleavage, the remaining fragment, termed C99, is cleaved by the γ-secretase complex, which results in the generation of Aβ. The exact site of the γ-secretase-induced cleavage of C99 can vary, resulting in Aβ peptides of different lengths [28, 29]. The most common forms, Aβ40 and Aβ42, possess 40 and 42 amino acids, respectively. Aβ42 is considered to be the most pathogenic in the development of AD [30]. Cleavage of the C99 fragment occurs in the trans-Golgi network followed by release of the peptides into the extracellular environment [26]. Generally, the balance between the production and clearance of Aβ leads to a standard plasma concentration of soluble Aβ protein. Consequently, the bloodstream becomes one of the major chronic supplies of Aβ peptides to the brain [31]. Both APP and its Aβ production pathway are involved in several cerebral physiological processes, such as neurotrophic activity, modulation of synaptic plasticity, neurogenesis, metal ion sequestration, antioxidant activity, and calcium homeostasis [32].

Several studies on AD have shown that APP and Aβ are strongly involved in the glucose regulation within the brain, and more specifically in neurons [33, 34]. Aβ42 can impair glucose transport directly by interacting with glucose transporter protein GLUT [35]. Aβ inhibits the glycolysis flux into neuronal cell lines by decreasing the activity of different enzymes, such as hexokinase [36], phosphofructokinase [37], or glyceraldehyde-3-phosphate dehydrogenase [38]. Also, APP and Aβ seem to play an important role in lipid regulation [39, 40] and may regulate the glucose cycle as a competitive inhibitor of insulin [41]. Studies on the isolated rat aorta have shown that Aβ has vasoconstrictive properties [42] and attenuates acetylcholine-mediated endothelium-dependent vasodilatation [43]. Studies in young Tg2576 mice expressing the APP Swedish mutation have demonstrated reduced cerebrovascular reactivity to endothelium-dependent vasodilators, an increased response to vasoconstrictors acting directly on vascular smooth muscle cells, as well as altered neurovascular coupling [44].

## Aβ Clearance

Aβ protein has an important physiological role, which can be dysregulated with aging or in AD [5, 32]. Impairment of the balance between Aβ production and clearance leads to an accumulation of Aβ within the brain parenchyma or in the vessel walls, which can generate neurodegenerative processes leading to dementia [45]. In the elderly human brain, Aβ deposition is more related to faulty Aβ clearance mechanisms (ca. 5%/h clearance for AD patients vs. ca. 8%/h clearance for control subjects, *p* < 0.03) than to overproduction of Aβ protein (ca. 7%/h of Aβ production for control subjects and AD patients) [14, 46, 47], although different forms of APP and Aβ overproduction can be the primary cause of Aβ accumulation in certain genetic forms of AD [48]. Aging is accompanied by decrease of Aβ clearance resulting from a lower rate of Aβ catabolism through reduced proteolysis, impaired transport across the blood–brain barrier (BBB), or impaired CSF transport [14, 46, 47]. To prevent Aβ aggregation and deposition, various physiological clearance mechanisms help to remove Aβ from the brain. These can be divided into the following three components: (1) transvascular clearance across the BBB, which is the most important clearance process (85%); (2) interstitial fluid (ISF) bulk flow and perivascular clearance through the CSF; and (3) uptake and enzymatic degradation by glia [49, 50]. These mechanisms are acting together to balance the cerebral Aβ concentration. During aging, the ratio between BBB clearance and ISF/CSF clearance decreases [50, 51]. The glymphatic system provides an additional perivascular Aβ clearance pathway, and its dysfunction could play an important role in the occurrence of Aβ deposits culminating into AD and dementia [14, 16]. This glymphatic pathway is thought to become drastically less effective by 80 to 90% with aging [14, 47].

### BBB-Mediated Aβ Clearance

The BBB is a highly selective membrane barrier that separates the circulating blood from the brain parenchyma in the central nervous system. The BBB is formed by endothelial cells that are connected by tight junctions, a thick basement membrane, and astrocyte endfeet. This exchange in the surface allows the selective passage or the selective transport of water, gases, lipid-soluble molecules, and glucose. It also prevents the entry of large molecules, including toxic compounds, into the brain [52]. About 85% of Aβ drainage is going through the BBB, partly via lipoprotein-related protein-1 (LRP-1) and an association between LRP-2 and apolipoprotein J (APOJ), the latter having more affinity for Aβ42 form [53, 54]. LRP-1 is expressed by vascular endothelial cells forming the BBB, pericytes, vascular smooth muscle cells, neurons, and astrocytes [55, 56]. Cerebral Aβ binds to surface LRP-1 on the abluminal side of the BBB and then crosses the BBB by transcytosis to be released in the bloodstream. Free circulating Aβ binds to soluble LRP-1 (sLRP-1) in plasma, and hepatic LRP-1 mediates systemic clearance of soluble LRP-1-Aβ complexes and free Aβ [53, 57]. In humans, sLRP-1 binds 70–90% of Aβ in plasma under normal conditions [58].

On the luminal membrane of cerebral vessels, free Aβ that escapes the sLRP-1 surveillance in the blood interacts with the receptor for advanced glycation end-products (RAGE). Aβ-RAGE interaction not only mediates transport of Aβ from blood to brain but also leads to the expression of adhesion molecules at the BBB and secretion of endothelin-1, which can directly suppress blood flow [59, 60]. Aβ surplus, leading to increased formation of Aβ-RAGE complex, may trigger a pathophysiological response characterized by a pro-inflammatory cascade and Aβ accumulation in both brain parenchyma and vasculature [61]. Also, Aβ oligomers interact with glial Toll-like receptors (TLRs) promoting the release of neurotoxic pro-inflammatory mediators [62].

RAGE expression is increased with normal aging and in AD, especially in the hippocampus. This suggests that a significant proportion of Aβ within the brains of AD patients is derived from the systemic circulation [63]. Moreover, reduced expression of LRP-1 has been reported during normal aging in rodents and non-human primates [61, 63] and in AD [64]. Increased ISF and CSF absorption through perivascular space, as outlined in the subsequent sections, may compensate the hampered Aβ clearance through the BBB.

### ISF Bulk Flow and Perivascular Aβ Clearance

Neural Aβ protein is released in the extracellular space and drained through the ISF. Brain and spinal cord ISF is derived from the blood, tissue metabolism, and CSF [16, 65]. The working of the ISF clearance system is comparable to that of the lymphatic system, in which solutes are drained through the basement membranes of capillaries and arteries. Injection studies have shown that tracers diffuse through the extracellular space of the brain parenchyma and cross basement membranes of capillaries to be drained out along basement membranes in the tunica media of arteries [66]. Previous studies have shown that ISF and solutes are subsequently drained to cervical lymph nodes [67], but this has been debated [68]. Aβ, which is drained through this system, can be used as a potential lymphatic drainage tracer in the human brain [6].

ISF drainage is directly linked to CSF production and movement, associated with vascular pulsation [69]. CSF is produced by the choroid plexus and passes through the ventricular system into the subarachnoid space. In humans, most of the CSF drains into the blood via arachnoid villi and granulations in the major venous sinus [70]. In recent years, studies have found that CSF can drain solutes, such as Aβ, out of the brain through the glymphatic system, along cranial nerves and possible lymphatic vessels in the dura [71–74]. In line with these findings, single-photon emission computed tomography combined with a systemic injection of radiolabeled Pittsburg Compound B (PiB) in rodents demonstrated that Aβ is drained out of the brain through the ISF/CSF in the nasal mucosa [75]. The authors of this study have also demonstrated that Aβ can be degraded in Aβ 1–19 or 1–20 by insulin-degrading enzyme within the CSF, which could facilitate Aβ clearance [75].

The abovementioned Aβ-eliminating mechanisms depend on the existence of vascular and extracellular matrix integrity, which degrades with ageing, increased vascular risks and vessel wall injury as found in CAA, non-amyloid vessel disease, and stroke [75].

### Enzymatic Aβ Clearance

Although the BBB and perivascular clearance pathways play a major role in Aβ level regulation, the Aβ amount is also regulated (i.e., degraded) by a large set of proteases with diverse characteristics [76, 77]. It has been demonstrated that insulin-degrading enzyme, neprilysin, and endothelin-converting enzymes 1 and 2 are strongly involved in Aβ clearance [78]. Furthermore, accumulation of Aβ, caused by proteolytic failure, has also been correlated with CAA [79]. Enhancing Aβ proteolysis prevents senile plaque formation and secondary pathology [80]. In contrast, depletion of these types of protease doubles the time of Aβ elimination from the ISF [81]. These proteases are the major target for Aβ proteolytic therapy, but other factors may also be of interest, such as metalloproteinases (e.g., MMP2, MMP9), serine proteases (e.g., plasmin), aspartyl proteases (e.g., cathepsin D, BACE), cysteine proteases (e.g., cathepsin B), threonine proteases (e.g., proteasome), or catalytic antibodies [81].

In the extracellular space, Aβ production is regulated by astrocytic and microglial LRP-1-related uptake [82]. The depletion of LRP-1 in astrocytes leads to a decrease of Aβ uptake and degradation by astrocytes. It has been demonstrated that LRP-1 depletion in rodents decreases Aβ degradation by astrocyte-related enzymes (MMPs or neprilysin) leading to an increase of Aβ protein in the cerebral parenchyma. The depletion of neprilysin in mice led to a 23% increase in the concentration of Aβ within the ISF, as well as an increase in Aβ half-life (1.7 vs. 2.1 h, *p* < 0.01) [81]. Indeed, AD is accompanied by a decrease of Aβ uptake by astrocytes and reduced production of astrocytic proteases caused by a lower expression of LRP-1 [65]. Astrocytes and smooth muscle cells also contribute to the uptake and degradation of Aβ in a process that may be mediated by the expression of the water channel protein aquaporin-4, which is considered a key component of the glymphatic system [14, 83].

Protein aggregates in cells, such as Aβ in neurons, are cleared by autophagy, a mechanism which is impaired in AD. Autophagy influences the secretion of Aβ into the extracellular space and thereby directly affects Aβ plaque formation [84]. By crossing APP transgenic mice with mice lacking autophagy in excitatory forebrain neurons (obtained by conditional knockout of autophagy-related protein 7), Nilsson and collaborators demonstrated that autophagy deficiency drastically reduces extracellular Aβ plaque burden. This reduction is due to the inhibition of Aβ secretion, which subsequently leads to aberrant intraneuronal Aβ accumulation in the perinuclear region. Moreover, autophagy deficiency-induced neurodegeneration is exacerbated by amyloidosis, which conjointly severely impairs memory [84].

### Aβ Clearance Through the Glymphatic System

Since the beginning of modern neuroscience research, the brain has been described as the only organ without an active lymphatic system. The role of CSF was described as a protective fluid against traumatic and mechanic shock. Perivascular space or Virchow-Robin’s space is known to guide the drainage of brain solutes from the brain to the lymphatic system. In 2012, Nedergaard and collaborators described new functions for this system in relation to glial cells, which was then termed the glymphatic system, emphasizing its lymphatic function in combination with glial aquaporin-4 (AQP4) water channels located on the astrocytic endfeet [85]. By injecting a tracer directly into the CSF of rats, they visualized its diffusion throughout the brain parenchyma and showed that the CSF penetration into the brain parenchyma is an active process through perivascular spaces [86]. These spaces were found around cerebral arteries and veins, with unique characteristics for CSF circulation. CSF enters the brain through arterial perivascular space and gets out through the venous perivascular space. This process would allow drainage of ISF and subsequent metabolization of waste products [12]. In 2015 and 2016, two studies also demonstrated the existence of true cerebral lymphatic vessels in the dura [13, 71], which may support the glymphatic system’s functions. Although the majority of studies of the glymphatic system have so far been done in rodents, there is evidence of the existence of a similar cerebral lymphatic system in the non-human primate [18] and the human brains [16, 87, 88].

The glymphatic system may play an important role in the transport of nutrients, such as glucose, from the blood to brain tissue [73], which can be impaired during physiological aging [14] and AD [15]. By calculating the MRI-detected contrast enhancement ratio between the olfactory bulbs and the cerebellum of mice after contrast agent injection in the cisterna magna, Gaberel and colleagues have observed that the glymphatic system is impaired after ischemic stroke (signal intensity ratio: 1.0 in controls vs. 0.7 after stroke, *p* < 0.05) and after subarachnoid hemorrhage (signal intensity ratio: 1.3 in controls vs. 0.5 after subarachnoid hemorrhage, *p* < 0.05) [17, 18]. Up to 40% of glymphatic system impairment may develop during aging, accompanied by a 27% reduction in the vessel wall pulsatility of intracortical arterioles, and widespread loss of AQP4 polarization along the penetrating arteries, which could contribute to Aβ deposition [14]. Also, deficiency of Aβ drainage through the glymphatic system could lead to spreading of Aβ deposits from the brain to the eye, possibly contributing to macular degeneration [89].

The glymphatic system appears particularly active during sleep [90, 91] controlling the lactate cycle and the drainage of the daily produced waste [91, 92]. However, this affirmation has been recently debated in two studies from different groups, attesting that the glymphatic system would be less effective in anesthetized mice, a condition that is not equivalent to real sleep [93, 94]. Sleep disorder has been reported to increase the risk for the development of AD by 1.5- to 2-fold. It has been considered as a risk factor for stroke, with strong correlation with Aβ deposition. In fact, a single night of sleep deprivation is correlated with higher morning Aβ levels [95, 96]. Impaired sleep is also a post-stroke symptom associated with poor functional recovery [97] and a consequence of AD [98]. How stroke, AD, and sleep disorder are related is yet unclear. Perivascular space modifications observed in CAA and AD could be key in this association [99, 100]. We can speculate that sleep disorder, associated with morphological perivascular space changes and glymphatic system deficiency, is a critical factor in the diagnosis of susceptible AD patients, with or without stroke event. Similarly, post-stroke disturbance of the perivascular space or the glymphatic system could enhance Aβ accumulation and deposition within the brain [16, 17]. Interestingly, a recent meta-analysis of a large cohort of stroke patients vs. controls shows that stroke is associated with long sleep and that people with 7 hours of daily sleep are less vulnerable to have a stroke [101].

## CAA Pathophysiology

The sporadic form of CAA is a cerebrovascular disease characterized by Aβ accumulation within the vessel walls of capillaries, arterioles, and small- and medium-sized arteries of the cerebral cortex, leptomeninges, and cerebellum [4]. The cascade of events promoting Aβ depositions in vessels and/or the brain parenchyma is not fully understood. Several studies support the concept that Aβ accumulation is not caused by an overproduction of Aβ, but due to a faulty Aβ clearance [83, 102, 103]. Three sources of Aβ can be detected, which are as follows: blood plasma, muscular layer of vessel walls, and neural cells [104]. The lack of CAA in transgenic mouse models with high Aβ plasma levels, and the lack of correlation between plasma Aβ and Aβ senile plaques, argue against plasma Aβ as the main source [105]. Nevertheless, circulating Aβ may enhance CAA [106]. Various transgenic mouse models with neuronal overexpression of APP support the suggestion that neuronally produced Aβ can give rise to CAA [103].

The main process leading to CAA appears to be faulty Aβ clearance. While parenchymal senile plaques associated with AD are more likely composed of Aβ42, Aβ40 is predominant in vascular deposition [22, 107]. Aβ42 is also more prone to aggregate in comparison to Aβ40 [108]. This corresponds with the notion that lower molecular weight protein (ca. 3 kDa) can cross the perivascular space and the BBB more easily than higher molecular weight proteins (ca. 40 kDa). This system is strongly dependent of AQP4-expression. In fact, absence of AQP-4 in mice leads to a reduction of 70% of brain metabolite waste drainage [85, 109]. CAA is strongly correlated with alterations of the vessel wall, which may lead to BBB breakdown and micro-bleeds [110]. The media and adventitia of the microvasculature, with infiltrated Aβ, may reveal loss of smooth muscle cells with replacement of the vascular media by amyloid and cellular thickening of the vessel walls [111, 112]. Perivascular leakage of blood compounds [113, 114], correlated with decreased expression of tight junction proteins and overexpression of matrix metalloproteases 2 and 9 [115], reflects BBB breakdown associated with advanced CAA.

The distribution of CAA is not equal throughout the brain. Pathological studies have shown that CAA with associated hemorrhagic stroke occurs predominantly in lobar and posterior areas [116, 117]. Recently, it has been shown that CAA also correlates with perivascular space enlargement, which affects ISF- and CSF-mediated Aβ clearance [99, 118]. Vascular amyloid deposition may disrupt perivascular space drainage via a perturbation in normal arteriolar pulsation [14, 48]. With aging, it is also possible that a decrease in ISF clearance predisposes the perivascular basement membranes to higher concentrations of deposited Aβ, hence exacerbating CAA processes. Aβ deposition may further impair or block perivascular drainage, leading to dilation of perivascular spaces, not only in the cortical grey matter but also in the underlying white matter, which itself is typically not directly affected by CAA. The enlarged perivascular space can reach several millimeters in diameter and may be visible with appropriate brain imaging [100, 118, 119].

## Stroke Impairs the Balance Between Aβ Production and Aβ Clearance

Data from animal models suggest that stroke can trigger accelerated Aβ deposition and CAA through interference with clearance pathways [120, 121]. Analyses of clinical cohort studies suggest that ischemic or hemorrhagic strokes are severe risk factors for development of cognitive decline and AD [11, 122, 123]. Post-stroke dementia develops in up to a third of patients within a year after stroke, which is strongly associated with advanced aging [124]. Memory disturbance can be due to the stroke event itself or due to AD, and the two kinds of cognitive impairments may also coexist [125].

Other risk factors affecting cerebrovascular integrity and involved in post-stroke dementia are atrial fibrillation, previous stroke event, myocardial infarction, diabetes mellitus, and previous transient ischemic attack [125]. Also, hypertension, which is a common risk factor for stroke and AD, has been shown to worsen Aβ-induced neurovascular dysfunction and to promote β-secretase activity. This leads to an increase of amyloidogenic APP processing, which may contribute to the pathogenic interaction between hypertension, stroke, and AD.

Despite increasing evidence of links between stroke and early AD in subsequent studies, the underlying mechanisms remain incompletely characterized. Based on existing literature, we here discuss and propose some major and minor hypotheses on how accelerated vessel amyloid deposition due to stroke can be one of the major mechanisms leading to post-stroke forms of dementia.

### Vascular Impairment

In cerebrovascular diseases, Aβ deposition in the vessels associated with CAA may further compromise vascular function, causing more severe cerebral blood flow (CBF) deficits during and after ischemia, thereby exacerbating cerebral infarction. Milner and collaborators have described that young APP mice, as compared to control mice, have a 46% larger infarct volume after experimental stroke, which is exacerbated with aging (85 ± 9 mm^3^ in APP mice vs. 46 ± 9 mm^3^ in control, *p* < 0.05) [126]. This process could become a vicious cycle. In mouse models of Aβ deposition, stroke has been shown to lead to (transient) accumulation of amyloid depositions in brain parenchyma as well as vessel walls [120, 121]. Furthermore, stroke-induced hypoxia can lead to overexpression of APP in vascular smooth muscle cells [127], which could expedite or exacerbate local CAA development and worsening of stroke outcome.

Ischemic and hemorrhagic stroke usually leads to disruption of the BBB [128]. Soluble Aβ proteins circulating in the plasma (mostly Aβ40) are for 70% bound to soluble LRP-1 [129]. This complex can then directly leak from the vascular compartment to the CSF or cerebral parenchyma after stroke [48, 53]. Furthermore, impaired LRP-1-mediated transcytosis across the BBB may lead to insufficient clearance of Aβ protein. Impaired LRP-1 and RAGE efficiency may also be due to hypoperfusion or hypoxia, which increase heparin-binding EGF-like growth factor (HB-EGF) mRNA [130]. Oxidated LRP-1 is no longer able to trap the Aβ protein from the parenchyma to release it in the intraluminal part of the vessels.

The increased presence of Aβ in the parenchyma triggers inflammatory and neurodegenerative processes [129], characteristic for CAA and AD [131, 132]. Based on this, Zlokovic (2008) have proposed a new model to explain how cerebrovascular diseases, such as stroke, can lead to AD [60]. Stroke-induced compromised BBB coincides with hypoperfusion and will lead to an accumulation of Aβ, which induces a neuroinflammatory response [133]. In an early phase, inadequate clearance of Aβ at the BBB may favor accumulation of neurotoxic Aβ oligomers in the brain ISF. Aβ oligomers combined with focal reduction in capillary blood flow can affect synaptic transmission, causing neuronal injury and recruitment of macrophages from the blood (monocytes) or within the brain (microglia). At an early stage, the BBB starts losing Aβ-clearing properties and the activated endothelium starts to secrete proinflammatory cytokines [134–136] and CBF-suppressing factors [129]. This may lead to synaptic dysfunction, accumulation of intracellular tangles, and activation of microglia, as well as acceleration of CAA [120]. In addition, activated microglial cells may develop intracellular Aβ depositions and/or release Aβ [137].

### Glymphatic System Impairment

Post-stroke loss of BBB integrity influences perivascular space integrity and glymphatic system efficiency [17, 18, 138]. Several studies, in animal models as well as in patients, have shown that impaired CSF and glymphatic clearance could contribute to accumulation and aggregation of waste products and other compounds in the CSF and brain parenchyma [139]. Some of those, such as lactate, tau, and Aβ, have been implicated in dementia development [90, 140, 141]. In addition to being a consequence of Aβ pathology, impairments in CSF clearance and the glymphatic system could promote Aβ accumulation [15, 142]. By using in vivo MRI of gadolinium-based contrast agent injected into the CSF, Gaberel and collaborators found that ischemic stroke in rodents leads to a decrease of CSF circulation through perivascular spaces, which could be explained by a decrease of arterial pulsation after artery occlusion [17, 143]. In the case of subarachnoid hemorrhage, the presence of blood and micro-thrombi inside the perivascular space mechanically blocks the circulation of the CSF, as demonstrated in mice [17] and in non-human primates [18]. These studies suggest that both ischemic and hemorrhagic stroke can directly affect the efficiency of the glymphatic system.

The perivascular space is one of the preferential sites for Aβ [16, 119]. Analysis of Aβ production and clearance in AD patients revealed that in the sporadic form of AD, Aβ40 and Aβ42 clearances through the perivascular space are reduced to 30% in comparison with healthy controls. The loss of AQP4 channels, one of the main components of the glymphatic system, on astrocyte end-feet with aging is associated with a decrease of ventricular CSF clearance correlated with an accumulation of Aβ within the brain [14, 51, 144], leading to AD [14, 16]. In human AD patients, the CSF production rate is 33% lower than in healthy patients, and an enlargement of the perivascular space has recently been demonstrated [99, 145]. This critical drop in CSF production would impact the efficacy of metabolic waste clearance through the PVS. As found in vitro, Aβ aggregation seems to be controlled by stochastic nucleation and dependent on Aβ concentration [146]. It is possible that the lack of Aβ clearance through the CSF leads to an increased concentration of Aβ in the brain parenchyma, which evolves in a cascade of aggregation when a critical concentration is reached. After ischemic or hemorrhagic stroke, the CSF circulation is also impaired. In a primate study using primate, it has been demonstrated that subarachnoid hemorrhage can block up to 30% of the CSF transport [18]. This lack of physiological circulation of the CSF in the PVS may mimic conditions found in AD patients and contribute to the accumulation of metabolic waste products and Aβ. Even though the lower CSF rate seems to be correlated with the presence of CAA, perivascular space enlargement is independent of the presence of CAA [142]. This enlargement may be linked to accumulation of Aβ in the perivascular space, leading to Aβ protein deposition-based “clot” formation [119]. Several pathological conditions, such as chronic arterial hypertension, atherosclerosis, CAA, and stroke, are associated with deformation of the perivascular space [147], which may affect clearance systems, such as the glymphatic system, leading to further enhancement of Aβ accumulation within the brain parenchyma, perivascular system, and vessel walls, and expediting dementia development.

Even when the glymphatic system is intact, toxic waste products (such as thrombin or iron) may spread from the primary lesion site to distant brain areas [148]. This could lead to characteristic secondary injuries, such as vasospasm, inflammation, micro-infarcts, delayed ischemia [149], and conceivably Aβ deposition in the presence of CAA or parenchymal Aβ accumulation.

Principal studies explaining the potential linkage between stroke, CAA, and dementia are presented in Table 1.

**Table 1.** Hypothetical mechanisms, described in the literature, which may explain the association between stroke, amyloid deposits, and early dementia

Mechanism hypothesis	References
Hemorrhagic and ischemic stroke induce Aβ deposits and CAA leading to dementia	Ellis et al. [10] Regan et al. [3] Gamaldo et al. [11] Pendlebury and Rothwell [124] Savva et al. [123] Cordonnier and van der Flier [9] Cerasuolo et al. [122]
Stroke induces Aβ accumulation within the cerebrovascular system by decreasing Aβ clearance	Garcia-Alloza et al. [121]
Stroke-induced hypoxia leads to an overexpression of APP	Rensink et al. [127] Ashok et al. [130]
Stroke-induced BBB breakdown allows blood Aβ infiltration within the brain parenchyma	Zlokovic et al. [60, 129] Yang and Rosenberg [128] Hawkes et al. [49] Ramanathan et al. [53]
Oxidated LRP-1 in AD or after stroke cannot interact properly with circulating Aβ	Donahue et al. [63] Ramanathan et al. [53] Ashok et al. [130] Liu et al. [82] Zlokovic et al. [60]
Post-stroke Aβ accumulation leads to inflammatory processes and neurodegeneration, typical in dementia	Zlokovic [131] Kinnecom et al. [132] Zlokovic et al. [60, 129]
Lack of Aβ-RAGE complexes after stroke leads to a pro-inflammatory cascade and Aβ accumulation, which can contribute to neurotoxicity	Deane et al. [61] Liu et al. [62]
Correlation between Aβ deposits and sleep disorder is a common risk factor for stroke and AD	Holth et al. [95] Ma et al. [96] Joa et al. [97]
CSF clearance and the glymphatic system are impaired with both stroke and AD, leading to accumulation of waste metabolites in the brain	Silverberg et al. [145] Weller et al. [16] Weller et al. [6] Kress et al. [14] Gaberel et al. [17] Peng et al. [15] Goulay et al. [18] Lundgaard et al. [90] Borwn et al. [19]
CAA and AD are associated with modifications in perivascular spaces, which can lead to flow disturbance, stroke, and Aβ accumulation	Mendelsohn and Larrick [92] Kress et al. [14] Hawkes et al. [49] Van Veluw et al. [100] Banerjee et al. [99] Charidimou et al. [118]

## Treatment Strategies

The studies in this review demonstrate that stroke can exacerbate CAA and/or enhance Aβ deposition, leading to cognitive decline and dementia. With regard to hemorrhagic and ischemic stroke, neuroprotective measures are crucial in order to prevent neuronal death and to avoid an increase of the BBB breakdown area [150]. First, glycemia should be controlled [151]. It has been recently recommended to limit glucose concentration in the blood to maximally 140–180 mg/dl because of neurotoxicity at higher levels [152]. Second, if possible, body temperature should be reduced. A high temperature has been associated with BBB breakdown and a worsening prognosis [153]. Finally, in case of acute ischemic stroke within 4.5–6 h, attempts to restore perfusion through thrombolysis or thrombectomy should be made [154]. Several preclinical trials have targeted the perivascular space and Aβ deposition to counteract CAA and neurodegeneration. Studies in aged TgSwDI mice, an APP-mutated strain developing Aβ deposits, suggested that counteracting the deleterious effects of Aβ after vascular depositions is not effective in reversing the neurovascular dysfunction associated with vascular smooth muscle cell damage caused by ageing and massive Aβ deposition [155]. Hence, strategies that focus on prevention or reduction of cerebrovascular injury and preservation of perivascular space integrity may be more effective in limiting vascular Aβ deposition.

Furthermore, the glymphatic system has recently been characterized as an additional potential therapeutic target [88]. This hypothesis is supported by a study using aquaporin-4 knockout (AQP4^−/−^) mice with hemorrhagic stroke, which showed significantly more gliosis, more severe neuroinflammatory patterns, and worse neurological outcome compared with wild-type mice [155]. This suggests that perivascular flow is critically involved in post-stroke cerebral tissue outcome and that the upkeep of this flow could be considered as a therapeutic strategy. In a rodent model of subarachnoid hemorrhage (SAH), it has been shown that disrupted CSF/ISF drainage can be cleared using the perivascular pathway with an intraventricular tissue-type plasminogen activator treatment [149, 156]. This approach has already been successfully applied in humans with hydrocephalus [157, 158]. This strategy alleviated histological injury and improved behavioral function after SAH [149, 157]. CSF drainage may also be applied to increase Aβ clearance in patients. However, in 2008, a clinical study on 215 patients with either mild dementia or Alzheimer’s disease did not show any benefit of CSF drainage through a low-flow ventriculoperitoneal shunt [159]. Alternatively, intraventricular fibrinolysis may more effectively remove sources of impaired CSF/ISF circulation, improve glymphatic system function, and increase Aβ clearance, but this needs to be confirmed in longitudinal studies. The potential risk that enhancement of CSF flow in the perivascular space could promote transport of Aβ depositions and other toxins to healthy brain areas [149] also requires further investigation.

Preventing the accumulation of Aβ in perivascular drainage pathways seems to be a valid therapeutic strategy in AD [16]. It has already been demonstrated that immunotherapy can remove established Aβ plaques from brain parenchyma by 90% [160, 161], relieving the restricted diffusion of solutes through the extracellular spaces, ultimately leading to cognitive recovery [129, 162]. However, this approach may increase presence of amyloid deposits within the vessel walls, promoting CAA and micro-bleeds [161]. Alternative strategies to reduce the amount of Aβ entering the perivascular space may be increasing the level of neprilysin in the brain or improving LRP-related clearance of Aβ into the blood [59]. From a therapeutic perspective, it has been shown in the past that upregulation of Aβ degradation appears to compare favorably to clearance-based therapeutics based on immunization against Aβ in mice [163, 164]. However, this approach has triggered deleterious immune-mediated reactions, brain edema, and immune-cell infiltration in patients [165]. Leissring and colleagues proposed that pharmacological upregulation of a single Aβ-degrading protease may achieve the same therapeutic benefit, while avoiding potentially adverse immune responses [79]. The finding that chronic upregulation of these proteases is not accompanied by detectable adverse effects in mice up to 15 months of age suggests that additional preclinical studies on the safety and efficacy of a proteolytic approach are worthy to carry out [79].

Park and colleagues recently examined the role of perivascular macrophages in the cerebrovascular action against Aβ, using an elegant method of macrophage depletion and bone marrow transplantation between different strains of mice [166]. They found that selective depletion of perivascular macrophages abrogates vascular oxidative stress and neurovascular dysfunction induced by Aβ, either administered to wild-type mice or produced endogenously in the brain of transgenic APP mice. Their observations suggest that perivascular macrophages are a main source of vascular reactive oxygen species responsible for Aβ-associated CBF alterations. Furthermore, in models of cerebral amyloidosis, perivascular macrophage depletion has been shown to reduce Aβ accumulation in cerebral blood vessels [167].

A recently published study highlights how the glymphatic system may be engaged as a therapeutic pathway. By applying a hypertonic solution, blood flow and perivascular flow could be elevated in wild-type and transgenic APP mice [168]. This points toward a promising therapeutic approach to restore glymphatic system efficiency and avoid Aβ accumulation within the vascular wall and the Virchow-Robin spaces in AD and after stroke.

### Other Mechanisms

While Aβ could be a key factor in the linkage between stroke and early dementia, other dementia ethiopathologies, such as tau protein aggregation and release of metal ions and free radicals, may be a sign. The hyperphosphorylation and abnormal aggregation of tau, combined with its decreased clearance, result in formation of neurofibrillary tangles, which exerts neurotoxicity in AD [140]. Furthermore, increase of oxidative or nitrosative stress, reduced antioxidant levels, and mitochondrial damage may also play major roles in the development and progression of AD [169]. Tau has been identified as a marker of poor outcome after stroke [170]. Its release may increase the excitotoxicity cascade through stimulation of glutamatergic receptors at the synapse and further progress neurodegeneration after stroke [171]. Free radicals and metals, released as a result of stroke injury, may induce disruption of several biomolecules, including DNA, which could contribute to early dementia [172, 173] and Aβ aggregation [174].

Thus, therapeutic strategies against free radical damage, protein oxidation, calcium and free radicals, tau and Aβ deposits, and phosphorylation may aid in the prevention of stroke-induced acceleration of dementia [175, 176].

## Conclusion

**Fig. 1.**
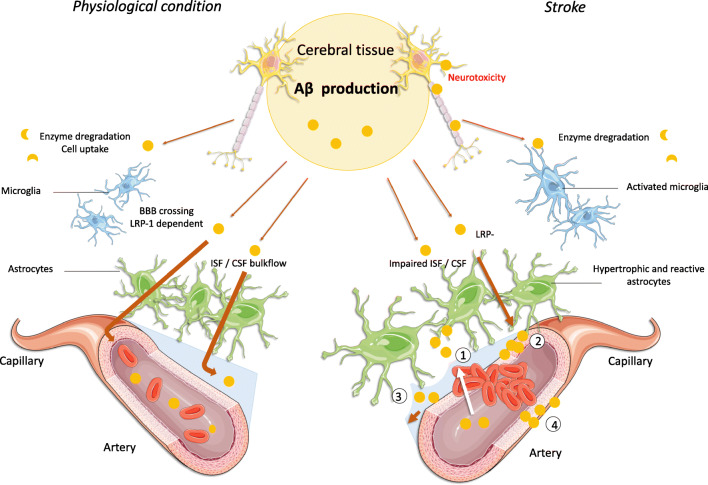
Aβ clearance under physiological conditions and after stroke. Under physiological conditions, Aβ is drained from the brain through a LRP-1 transcytosis pathway, which releases Aβ into the bloodstream; through the interstitial fluid/cerebral blood flow, i.e., perivascular (or glymphatic) Aβ clearance; and/or through enzymatic degradation and cellular uptake. After an ischemic stroke, these pathways may be impaired: (1) Blood–brain barrier leakage and astrocyte end-feet detachment, allowing circulating Aβ to enter the brain parenchyma. (2) Oxidation of the LRP-1 receptor, leading to an impairment to bind Aβ and to shuttle Aβ from the parenchyma to the luminal side of vessels. (3) Disrupted perivascular space circulation. (4) Hypoxia leading to an overproduction of Aβ in the vessels’ muscular layer. These processes can lead to Aβ deposition within the brain and cerebral vessels, leading to early amyloid angiopathy and neurotoxicity involved in dementia and Alzheimer’s disease

CAA and impairment of perivascular spaces are not only risk factors, but also consequences, of both AD and stroke. Our review highlights the tight link between cerebrovascular disease and dementia, in which stroke may have a “snowball effect” by enhancing and exacerbating CAA, leading to accelerated AD. This process appears particularly associated with impaired Aβ clearance pathways caused by cerebrovascular insufficiency, BBB breakdown, and/or perivascular space impediments (including glymphatic system dysfunction), as illustrated in Fig. 1. These factors also provide potential therapeutic targets that should be further assessed in future preclinical and clinical studies aiming to reduce the “snowball effect” of stroke on AD development.
